# In Vitro Assessment of the Combined Activity of Amphotericin B and Cu^2+^-1,10-Phenanthroline-5,6-dione Coordination Compound Against *Leishmania amazonensis* Promastigotes

**DOI:** 10.3390/tropicalmed11010004

**Published:** 2025-12-24

**Authors:** Simone Santiago Carvalho de Oliveira, Débora Duarte Batista, Michael Devereux, Malachy McCann, Christiane Fernandes, André Luis Souza dos Santos, Marta Helena Branquinha

**Affiliations:** 1 Laboratório de Estudos Avançados de Microrganismos Emergentes e Resistentes (LEAMER), Departamento de Microbiologia Geral, Instituto de Microbiologia Paulo de Góes (IMPG), Universidade Federal do Rio de Janeiro (UFRJ), Rio de Janeiro 21941-901, Brazil; simonesantiagorj@yahoo.com.br (S.S.C.d.O.); deboraduartefab@gmail.com (D.D.B.); 2 The Centre for Biomimetic & Therapeutic Research, Focas Research Institute, Technological University Dublin, D08 CKP1 Dublin, Ireland; michael.devereux@tudublin.ie; 3 Chemistry Department, Maynooth University, W23 F2H6 Maynooth, Ireland; malachy.mccann@nuim.ie; 4 Departamento de Quimica, Universidade Federal Santa Catarina (UFSC), Florianópolis 88035-972, Brazil; christiane.horn@ufsc.br; 5 Programa de Pós-Graduação em Bioquímica, Instituto de Química, Universidade Federal do Rio de Janeiro (UFRJ), Rio de Janeiro 21941-909, Brazil

**Keywords:** leishmaniasis, Cu^2+^-phendione, Amphotericin B, drug combination

## Abstract

Leishmaniasis is a severe parasitic disease transmitted by sandflies that affects both humans and animals, with clinical manifestations ranging from cutaneous lesions to life-threatening visceral involvement. Current treatments are limited by toxicity, high cost, and the emergence of drug-resistant strains, underscoring the need for safer and more effective therapeutic strategies. In this study, we investigated the antiparasitic potential of combining Amphotericin B, a drug commonly used for leishmaniasis treatment, with 1,10-phenanthroline-5,6-dione (phendione) coordinated to copper (Cu^2+^-phendione), an experimental coordination compound, against *Leishmania amazonensis* promastigotes. The combination markedly impaired parasite proliferation, disrupted ultrastructural integrity, and interfered with metabolic activity. Mechanistic analyses revealed the presence of autophagosomes and pronounced mitochondrial alterations in treated parasites, suggesting the induction of cellular stress and the disruption of essential survival pathways. In addition, the treatment reduced the association index with THP-1 cells, indicating a decrease in parasite infectivity. Collectively, these findings demonstrate that the combination of Cu^2+^-phendione and Amphotericin B exerts potent antiparasitic effects through multiple mechanisms. Our results also showed that Cu^2+^-phendione combined with AmB displayed an additive effect, although the isobologram suggested that certain ratios approached synergy. The results support the potential of this combination as a novel chemotherapeutic approach against leishmaniasis and provide a basis for future in vivo studies to evaluate safety, efficacy, and optimal dosing strategies.

## 1. Introduction

Leishmaniasis is one of the most relevant neglected tropical diseases (NTDs) in global public health and is considered the second most lethal parasitic infection after malaria [1,2,3]. Millions of people are estimated to be at risk, especially in tropical and subtropical regions, where factors such as poverty, inadequate sanitation, and limited access to health services favor transmission [4,5]. The disease is caused by protozoa of the genus *Leishmania*, transmitted by infected sandflies, and can be present clinically in three main forms: cutaneous (CL), characterized by ulcerated skin lesions; mucocutaneous (LMC), which affects the nasal and oropharyngeal mucosa; and visceral (VL), the most severe form, associated with prolonged fever, hepatosplenomegaly and significant weight loss, and which can be fatal if left untreated [2,5]. Clinical heterogeneity is influenced by both the parasite species and the host’s immune response. In some individuals, infection remains asymptomatic, whereas in others, it progresses to severe and debilitating disease [6,7]. This diversity of clinical manifestations, combined with the complexity of the parasite’s life cycle and its capacity for intracellular adaptation, underscores the challenges associated with the diagnosis, treatment, and control of leishmaniasis [6,7].

The therapeutic management of leishmaniasis remains limited and is associated with multiple challenges. This situation is exacerbated by the absence of an effective vaccine for human leishmaniasis capable of preventing disease and blocking transmission [6], leaving pharmacological treatment as the only currently available option. However, many of the traditional drugs are highly toxic, difficult to administer, and expensive, and their effectiveness is increasingly compromised by the emergence of parasite resistance [2,8,9]. Among the available options, Amphotericin B (AmB) remains one of the drugs of choice. It exhibits high affinity for parasite ergosterol, leading to membrane disruption and rapid parasite death, besides inducing ROS production. It has proven efficacy in visceral leishmaniasis, with additional evidence of activity against cutaneous forms [10,11]. Despite its effectiveness and low propensity for resistance, AmB is associated with serious adverse effects, particularly nephrotoxicity, which limits its clinical use and often requires hospitalization and close monitoring. Liposomal formulations offer reduced toxicity, but their high cost limits widespread use, especially in endemic regions [11,12]. Thus, although AmB is one of the most effective drugs currently available, its clinical application remains constrained, which reinforces the urgent need for new therapeutic alternatives.

In this context, metal coordination compounds have been investigated as potential antileishmanial agents. Among them, 1,10-phenanthroline-5,6-dione (phendione) coordinated to copper (Cu^2+^-phendione) stands out for its significant activity against various *Leishmania* species [13,14,15]. Evidence indicates that Cu^2+^-phendione can negatively modulate the expression of the metalloprotease gp63, a surface protein essential for parasite adhesion, invasion, and establishment of infection in host cells [16]. Furthermore, this compound disrupts the parasite’s redox homeostasis, inducing oxidative stress and mitochondrial dysfunction that compromise parasite viability [14,15].

One promising strategy involves the combination of conventional drugs with new compounds to enhance therapeutic efficacy and reduce side effects [17,18,19]. Combination therapy, which consists of the simultaneous or sequential administration of two or more drugs with distinct mechanisms of action, has emerged as a viable alternative to conventional monotherapy. This approach can improve efficacy, reduce toxicity, and help prevent the development of drug resistance [17,20]. Several innovative combination strategies have been investigated for the treatment of leishmaniasis. Notable examples include the combination of nimodipine and pentamidine, which demonstrated synergistic activity [21]; the combination of nitazoxanide with AmB, Glucantime^®^ or miltefosine, which showed additive effects against *Leishmania infantum* without inducing macrophage toxicity [22]; and the combination of paromomycin with sodium stibogluconate, which resulted in greater efficacy and lower toxicity than monotherapy [23]. Nanotechnology-based approaches have also gained attention. For instance, AmB combined with doxorubicin in nanomicelles enhanced treatment against visceral leishmaniasis, reduced toxicity, and exhibited excellent biosafety and biocompatibility profiles [24]. Iron oxide nanoparticles coated with AmB and chitosan demonstrated improved activity against both promastigote and amastigote forms of *Leishmania* spp. compared with AmB alone, while also decreasing toxicity in macrophages [25]. Natural compounds have likewise been explored in combination therapies. Curcumin combined with miltefosine increased efficacy against visceral leishmaniasis [26], whereas miltefosine combined with antimicrobial peptides produced a synergistic effect on intracellular forms of *L. infantum* [27]. In addition, the combination of miltefosine with lopinavir showed additive and potentially beneficial effects in HIV-*Leishmania* coinfections [28].

Thus, the combination of established drugs, such as AmB, with innovative molecules, such as Cu^2+^-phendione, opens new perspectives for the treatment of leishmaniasis. When combined with other compounds, AmB may be used in lower doses and potentially reduce treatment-related toxicity while maintaining its therapeutic efficacy. In parallel, the pleiotropic mechanism of Cu^2+^-phendione, some of which differ from the targets of AmB, enhances the overall effect of the combination and decreases the likelihood that single-point mutations will confer clinically relevant resistance [29,30]. Therefore, the search for more effective and less toxic therapeutic options remains essential to overcoming the long-standing challenges associated with leishmaniasis. In this context, the present study investigated the effects of the metal coordination compound derived from 1,10-phenanthroline, Cu^2+^-phendione, in combination with AmB, on *Leishmania amazonensis* promastigotes, one of the etiological agents of cutaneous leishmaniasis in Brazil.

## 2. Materials and Methods

### 2.1. Parasites and Cultivation

*Leishmania amazonensis* (MHOM/BR/PH8) was provided by *Leishmania* Type Culture Collection-LTTC-WDCM 731 (Fundação Oswaldo Cruz, Rio de Janeiro, Brazil). Promastigotes were cultured in Schneider’s insect medium (Sigma-Aldrich, St. Louis, MO, USA), pH 7.2, containing 10% heat-inactivated fetal bovine serum (FBS) (Cultilab, São Paulo, Brazil) at 28 °C.

### 2.2. Test Compounds

[Cu(phendione)_3_](ClO_4_)_2_·4H_2_O (Cu^2+^-phendione) was prepared in accordance with published methods [13]), and a 1 mM stock solution was prepared in dimethylsulfoxide (DMSO; Sigma-Aldrich, St. Louis, MO, USA). AmB was purchased from Sigma-Aldrich (St. Louis, MO, USA), and a 5 mM stock solution was also prepared in DMSO.

### 2.3. Effect of AmB and Cu^2+^-Phendione on the Growth Rate of Promastigotes

Promastigotes were counted using a Neubauer chamber and resuspended in fresh medium at a final concentration of 5 × 10^5^ viable promastigotes per milliliter. The viability was assessed by mobility and a lack of staining after challenging with Trypan blue. AmB was added to the culture at final concentrations ranging between 0.05 and 0.25 µM, and Cu^2+^-phendione was added to the culture at final concentrations ranging from 1 to 40 nM. After 24, 48 and 72 h of incubation at 28 °C, the number of viable parasites was estimated. The 50% inhibitory concentration (IC_50_), i.e., the drug concentration that caused a 50% reduction in survival/viability, was determined after 48 h by a linear regression analysis, by plotting the log number of promastigotes versus drug concentration using GraphPad Prism 8 computer software. As an additional control group, DMSO dilutions equivalent to those used for the highest drug concentrations were tested in parallel, and the solvent showed no effect on parasite growth behavior.

After establishing the IC_50_/48 h value of both compounds, promastigotes were treated with a combination of these drugs in 24-well plates. For this, promastigotes (5 × 10^5^ viable cells/mL) were incubated in the presence of the ¼ × IC_50_/48 h value of AmB and/or the ½ × IC_50_/48 h value of Cu^2+^-phendione. The number of viable motile parasites was estimated daily by counting the flagellates in a Neubauer chamber up to 72 h.

### 2.4. Parasite Treatment Protocol

For the experiments described in topics 2.5–2.12, *L. amazonensis* promastigotes (5 × 10^5^ cells/mL) were incubated for 48 h in the absence (control system) and in the presence of either ½ × IC_50_ Cu^2+^-phendione, ¼ × IC_50_ AmB or the combination of both fractions. In these experiments, the influence of the test compounds was tested on the (i) morphology, (ii) ultrastructure, (iii) membrane permeability, (iv) mitochondrial activity, (v) reactive oxygen species (ROS) production and (vi) adhesion/interaction of promastigotes with THP-1 cells. This combination was selected based on previous findings from our research group, which showed that *L. amazonensis* promastigotes exposed to these individual fractional concentrations did not exhibit significant alterations in cell viability, plasma membrane integrity, or mitochondrial activity [14,31]. These fractions were therefore chosen to ensure that each compound, when used alone, would produce minimal biological impact, allowing us to more clearly assess the effects resulting from their combined action.

### 2.5. Morphology and Morphometry

To detect morphological alterations, promastigotes were washed three times with phosphate-buffered saline (PBS; pH 7.2), fixed with methanol for 10 min, stained with Giemsa and observed under a Zeiss microscope (Axioplan, Oberkochen, Germany). In parallel, parasites treated under the same conditions were analyzed by flow cytometry (FACSCalibur, BD Bioscience, Franklin Lakes, NJ, USA) using a two-parameter histogram of forward scatter (FSC) to measure cell size and side scatter (SSC) to measure granularity/complexity. In each FACS experiment, 10,000 events were analyzed.

### 2.6. Scanning Electronic Microscopy (SEM)

Promastigotes were fixed with 2.5% glutaraldehyde in 0.1 M cacodylate buffer, pH 7.2 for 40 min at 25 °C. After this step, parasites were washed in cacodylate buffer and post-fixed with a solution of 1% OsO_4_, 0.8% potassium ferrocyanide and 5 mM CaCl_2_ in the same buffer for 20 min at 25 °C. Subsequently, promastigotes were dehydrated in rising acetone concentrations (30–100%) for 15 min each. Cells were dried on critical points and metallized with gold (25 nm) at 40 mA for 160 s and then observed in a scanning electron microscope (Thermo Quattro S, Thermo Fisher Scientific, Waltham, MA, USA).

### 2.7. Transmission Electron Microscopy (TEM)

Promastigotes were washed in PBS and fixed overnight at 4 °C in 2.5% glutaraldehyde in 0.1 M cacodylate buffer, pH 7.2. Cells were then washed in cacodylate buffer and post-fixed for 1 h in 0.1 M cacodylate buffer containing 1% osmium tetroxide, 0.8% potassium ferrocyanide and 5 mM CaCl_2_. After washing in the same buffer, cells were dehydrated in acetone and embedded in Epon resin. The ultra-thin sections were mounted on 300 mesh grids, stained with uranyl acetate and lead citrate and observed under a Zeiss 900 transmission electron microscope (Zeiss, Oberkochen, Germany).

### 2.8. Cell Membrane Integrity

Promastigotes were washed in PBS and incubated with 10 μM propidium iodide (PI; Sigma-Aldrich, St Louis, MO, USA)), a DNA-binding vital dye, for 30 min at room temperature, protected from light. The parasites were washed in PBS and their fluorescence was quantified on a flow cytometer (FACSCalibur, BD Bioscience, Franklin Lakes, NJ, USA) equipped with a 15 mW argon laser emitting at 535 nm. The mapped population (10,000 events) was analyzed for log red fluorescence using a single-parameter histogram. Cells treated with 4% paraformaldehyde were used as a positive control of passive PI incorporation.

### 2.9. Mitochondrial Metabolism

MTT [3-(4,5-dimethylthiazol-2-yl)-2,5-diphenyltetrazolium bromide] (Sigma-Aldrich, St Louis, MO, USA) assay was employed for mitochondrial metabolism testing in sterile 96-well plates [32]. Promastigotes were resuspended to a final concentration of 10^6^ parasites/mL and then MTT solution (5 mg/mL in PBS, 50 μg/well) was added prior to incubation for 3 h in the dark at 37 °C. After centrifugation at 300× *g* for 8 min, the supernatant was removed, the pellet was dissolved in 200 μL of DMSO and absorbance was measured in a microplate reader (SpectraMax Gemini 190, Molecular Devices, San Jose, CA, USA) at 490 nm.

### 2.10. Mitochondrial Transmembrane Electric Potential

The mitochondrial transmembrane electric potential (ΔΨm) was investigated using the JC-1 fluorochrome (Sigma-Aldrich, St. Louis, MO, USA), which is a lipophilic cationic mitochondrial vital dye that becomes concentrated in the mitochondrion in response to ΔΨm. Thus, the fluorescence of JC-1 is considered an indicator of an energized mitochondrial state, and it has been used to measure the ΔΨm in *Leishmania* [33]. Promastigotes were harvested after 48 h, washed with PBS and added to a reaction medium containing 125 mM sucrose, 65 mM KCl, 10 mM HEPES/K^+^, pH 7.2, 2 mM Pi, 1 mM MgCl_2_ and 500 µM EGTA. To evaluate the ΔΨm for each experimental condition, 2 × 10^5^ parasites were incubated with 10 µg/mL JC-1 for 40 min, with readings made every minute using a microplate reader (SpectraMax Gemini 190, Molecular Devices, San Jose, CA, USA). The relative ΔΨm value was obtained by calculating the ratio between the reading at 590 nm and the reading at 530 nm (590:530 ratio) using an excitation wavelength of 480 nm. Cells were also incubated in the presence of carbonyl cyanide 4-(trifluoromethoxy)phenylhydrazone (CCCP) at 1 µM, a mitochondrial protonophore, as a positive control of the depolarization of the mitochondrial membrane. CCCP at a concentration of 2 µM was also added at the end of all experiments to abolish ΔΨm. This allowed comparison of the magnitude of ΔΨm under the different experimental conditions.

### 2.11. ROS Production

Promastigotes were washed and resuspended in 500 µL of PBS and incubated with the cell-permeable probe dichlorofluorescein (H2DCFDA; Sigma-Aldrich, St. Louis, MO, USA) at 40 µM for 30 min in the dark. Then, cells were harvested at 500× *g*/5 min, resuspended in PBS, and analyzed by flow cytometry (FACSCalibur, BD Bioscience, Franklin Lakes, NJ, USA). Cells treated with hydrogen peroxide at 1 mM were used as a positive control [34]. The mapped population (10,000 events) was analyzed for log green fluorescence using a single-parameter histogram, and the results were expressed as the %FC.

### 2.12. Leishmania–Macrophage Interaction

Human leukemia monocytic cell line (THP-1) was maintained in 25-cm^2^ tissue culture flasks with RPMI 1640 medium (Sigma-Aldrich, St. Louis, MO, USA) supplemented with 10% FBS at 37 °C in an atmosphere containing 5% CO_2_. The culture medium was exchanged every three days. For interaction experiments, THP-1 cells in 24-well plates (2 × 10^5^ cells/well) were differentiated into macrophages by treatment with phorbol-12-myristate-13-acetate (PMA; 40 ng/mL) (Sigma-Aldrich, St. Louis, MO, USA) for 48 h. Then, the plates were washed twice with sterile PBS to remove PMA and a new RPMI 1640 medium was added [35]. Differentiated cells, used in all experiments, showed similar morphological changes and the ability to adhere to the culture plates as macrophages.

THP-1 differentiated cells (2 × 10^5^ cells) were placed in 24-well culture plates containing sterile cover glasses in each well. Then, *L. amazonensis* promastigotes (5 × 10^5^) were washed with PBS and pre-treated or not with each system described previously for 4 h, the time interval in which more than 95% of the parasites were viable, as judged by their morphology and motility. Mammalian cells were then infected with promastigote forms at a ratio of 10:1 (parasites/macrophage) for 24 h at 37 °C in a 5% CO_2_ atmosphere, after which monolayers were washed with PBS to remove unbound parasites. The coverslips were then fixed in methanol, stained with Giemsa and dehydrated in acetone solutions progressively replaced by xylol. The percentage of infected mammalian cells was determined by randomly counting at least 200 cells in each of the duplicated coverslips. The association index was obtained by multiplying the percentage of infected cells by the number of amastigotes per infected cell.

### 2.13. Combination Assay of AmB and Cu^2+^-Phendione

The interaction between AmB and Cu^2+^-phendione was evaluated in vitro using a modified isobologram method [28,36,37] in 96-well plates. For this, promastigotes (5 × 10^5^ viable cells/mL) were incubated in Schneider’s medium at 28 °C for 48 h. To define the maximum concentrations of each individual drug, the previously established IC_50_ values were used, ensuring that the IC_50_ of each compound would fall at about the fourth point of the serial dilution. The highest concentration of the solutions was prepared in proportions of 5:0, 4:1, 3:2, 2:3, 1:4, and 0:5 of Cu^2+^-phendione and AmB, which were subsequently subjected to serial dilutions (base 2) up to the seventh well of the microplate, in triplicate. After 48 h of incubation, the IC_50_ value was determined for the compounds in combination, in each proportion. Viability was evaluated by Resazurin dye/Alamar blue (7-hydroxy-3H-phenoxazin-3-one-10-oxide; Sigma-Aldrich, St. Louis, MO, USA) assay, which was added to a final concentration of 0.0125% in PBS. After a 4 h incubation at room temperature, parasites were analyzed at a microplate reader (SpectraMax Gemini 190, Molecular Devices, San Jose, CA, USA) using a pair of 590 and 544 nm as emission and excitation wavelengths, respectively [38]. Control values were considered as 100% of viability.

The fractional inhibitory concentration (FIC) was determined for both AmB and Cu^2+^-phendione by dividing the IC_50_ value of each compound in combination by the IC_50_ value of each compound alone. The sum of the FICs (ΣFIC) for each ratio was determined, and the arithmetic mean of ΣFIC in each combination was calculated to infer the type of interaction between the compounds based on the following values: synergic (ΣFICs ≤ 0.5), additive (0.5 < ΣFICs ≤ 2.0) or antagonic (ΣFICs > 2) [39]. For each drug combination and susceptibility assay, three independent experiments were conducted. FIC values of each drug ratio were used to build an isobologram.

### 2.14. Statistical Analysis

All experiments were performed in triplicate in three independent experimental sets. Data were analyzed by one-way ANOVA followed by Tukey’s post hoc test using GraphPad Prism 4.0 software (Prism, version 8.0; GraphPad Software; San Diego, CA, USA, 2018). Descriptive analysis, including mean and standard deviation, was used to evaluate numerical data. *p* values of 0.05 or less were considered statistically significant.

## 3. Results

### 3.1. Effect of Cu^2+^-Phendione and AmB on the Proliferation Rate of L. amazonensis Promastigotes

Initially, we evaluated the susceptibility of *L. amazonensis* promastigotes to the metal complex Cu^2+^-phendione and to AmB. For this analysis, IC_50_ values were determined as the concentrations required to reduce cell viability by 50%. Both compounds inhibited parasite proliferation in a dose-dependent manner, with IC_50_ values of 8.37 nM for Cu^2+^-phendione and 0.14 μM for AmB after 48 h.

**Figure 1:**
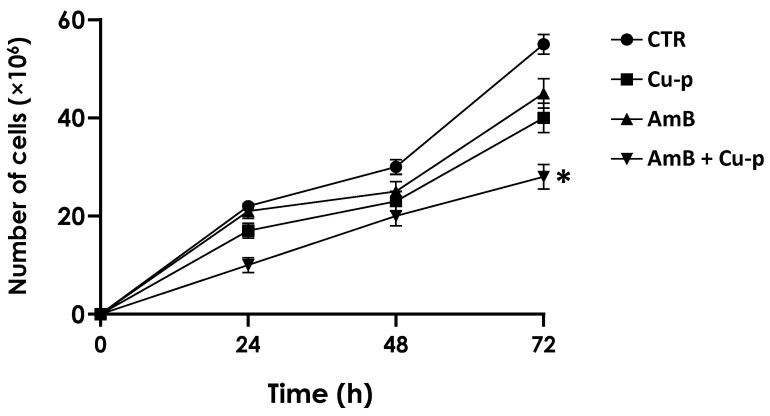
Effects of the combination of ½ × IC_50_ Cu^2+^-phendione (Cu-p) and ¼ × IC_50_ Amphotericin B (AmB) on the proliferation rate of *L. amazonensis* promastigotes. The parasites were cultured at 28 °C in the absence (CTR) or in the presence of both drugs in the combination cited above (AmB + Cu-p). In addition, the growth rate of promastigotes was monitored in the presence of either the ¼ × IC50 value of AmB or the ½ × IC_50_ value of Cu-p. The number of viable cells was estimated daily up to 72 h in a Neubauer chamber. Results represent mean ± standard deviation of three independent experiments performed in triplicate. Asterisk highlights the significantly different growth rate of the parasites in the presence of AmB + Cu-p in all time intervals when compared to the control (*p* ˂ 0.05).

We next assessed the effects of the combination of both compounds on promastigote proliferation over 72 h. For this purpose, a combination consisting of ¼ × IC_50_ AmB and ½ × IC_50_ Cu^2+^-phendione was used in this study. This combination significantly reduced the proliferation of *L. amazonensis* promastigotes at all time points compared with control cells (Figure 1) and was therefore selected for the subsequent experiments.

### 3.2. Effects on Morphology

**Figure 2:**
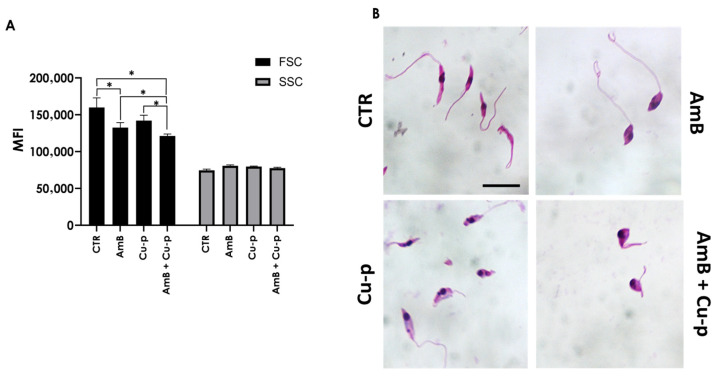
Effects of the combination of ½ × IC_50_ Cu^2+^-phendione (Cu-p) and ¼ × IC_50_ Amphotericin B (AmB) on the morphology of *L. amazonensis* promastigotes. (**A**) Promastigotes were treated or not (CTR) with either Cu-p, AmB or with the combination (AmB + Cu-p) for 48 h and analyzed by flow cytometry. Cell size (FSC) and granularity (SSC) were expressed as mean fluorescence intensity (MFI). Data are presented as mean ± standard deviation from three independent experiments performed in triplicate, with 10,000 cells analyzed. Results with *p* < 0.05 (*) were considered significant. (**B**) In parallel, promastigotes were fixed with methanol, stained with Giemsa, and analyzed by light microscopy. Control promastigotes display the kinetoplast (k) at the anterior region, a central nucleus (n), and a flagellum (f) connected to the cell body. Scale bar = 5 μm.

Treatment of *L. amazonensis* promastigotes with ½ × IC_50_ Cu^2+^-phendione alone did not induce morphological alterations compared with untreated cells, as assessed by flow cytometry (Figure 2A). In contrast, exposure to the AmB fraction (¼ × IC_50_ value) resulted in a significant reduction in cell size. The simultaneous application of Cu^2+^-phendione and AmB produced a significant effect not only relative to the control but also compared with each fraction tested individually (Figure 2A). No significant alterations in granularity were observed under any treatment conditions (Figure 2A). Giemsa staining corroborated these findings: untreated parasites displayed the typical promastigote morphology, characterized by an elongated body, anteriorly positioned kinetoplast, and long flagellum (Figure 2B). Cells treated with Cu^2+^-phendione showed no noticeable morphological changes, whereas AmB-treated cells exhibited reduced cell size and rounding of the cell body. In contrast, parasites exposed to the combined treatment displayed more pronounced morphological alterations than those observed with either compound alone (Figure 2B).

**Figure 3:**
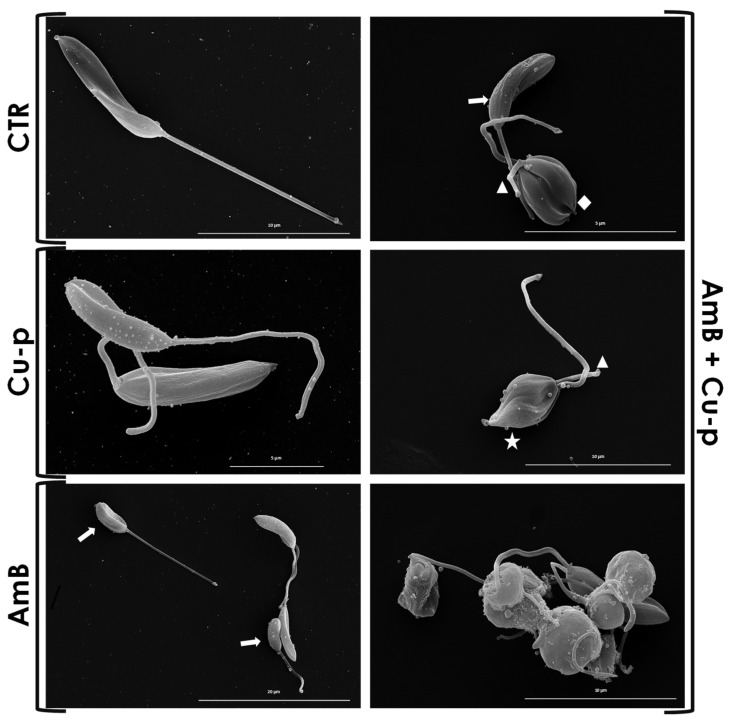
Scanning electron microscopy (SEM) of *L. amazonensis* promastigotes after treatment with ½ × IC_50_ Cu^2+^-phendione (Cu-p), ¼ × IC_50_ Amphotericin B (AmB), or the combination of both compounds (Amb + Cu-p). Control parasites (CTR) displayed normal morphology with an elongated cell body and long flagellum. Promastigotes treated with ½ × IC_50_ Cu-p showed no significant structural changes. Treatment with ¼ × IC_50_ AmB induced minor morphological alterations, such as shortening of the cell body (white arrows). Parasites treated with the drug combination exhibited diverse morphological alterations, including shortened cell bodies (white arrows), rounded cells (diamond), reduction or loss of the flagellum (white arrowhead) and fusiform bodies (star), as well as the formation of cell aggregates.

Scanning electron microscopy revealed that untreated parasites displayed normal morphology, characterized by an elongated cell body and an extracellular flagellum (Figure 3, CTR). In contrast, cells treated only with the Cu^2+^-phendione fraction retained the typical promastigote morphology (Figure 3, Cu-p), whereas parasites exposed solely to the AmB fraction exhibited minor alterations, such as a slight shortening of the cell body (Figure 3, AmB). More pronounced changes were observed in parasites treated with the combination of compounds, including cell body shortening and rounding, the appearance of fusiform forms, reduction or loss of the flagellum, and the formation of cellular aggregates (Figure 3, AmB + Cu-p). These findings demonstrate that the drug combination (¼ × IC_50_ AmB and ½ × IC_50_ Cu^2+^-phendione) induces significant morphological alterations in *L. amazonensis* promastigotes.

### 3.3. Effects on Ultrastructure

**Figure 4:**
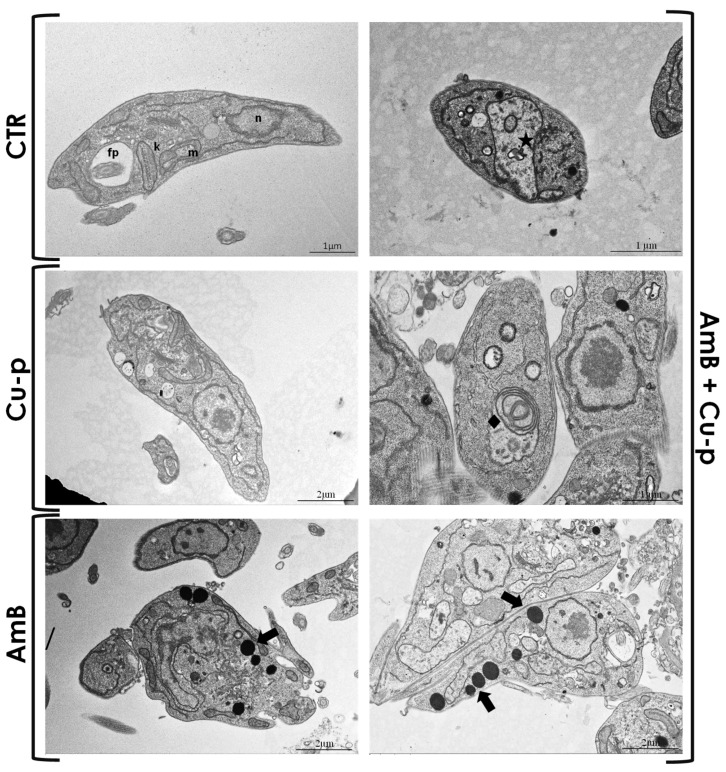
Transmission electron microscopy (TEM) of *L. amazonensis* promastigotes after treatment with ½ × IC_50_ Cu^2+^-phendione (Cu-p), ¼ × IC_50_ Amphotericin B (AmB), or the combination of both compounds (Amb + Cu-p). Untreated control cells (CTR) maintained the typical promastigote morphology, with a well-defined nucleus (n), kinetoplast (k), mitochondrion (m) and flagellar pocket (fp), without evident ultrastructural changes. *L. amazonensis* promastigotes treated with the Cu^2+^-phendione fraction showed no significant ultrastructural alterations. Treatment with the AmB fraction induced minor cellular changes, such as cytoplasmic lipid accumulation (black arrow). In contrast, promastigotes treated with the drug combination displayed multiple ultrastructural alterations, including mitochondrial swelling and disorganization (star), accumulation of lipid bodies (black arrows) and autophagosome-like structures (diamond).

Transmission electron microscopy revealed distinct ultrastructural alterations in *Leishmania* promastigotes (Figure 4). Untreated control cells (Figure 4, CTR) maintained the typical promastigote morphology, with no evident ultrastructural abnormalities. Treatment with ½ × IC_50_ Cu^2+^-phendione (Figure 4, Cu-p) did not induce significant morphological changes, as the cytoplasm and organelles remained intact, indicating minimal effect at this concentration. Exposure to ¼ × IC_50_ AmB (Figure 4, AmB) produced mild alterations, including the appearance of lipid droplets, suggesting the onset of cellular stress. In contrast, the combined treatment with Cu^2+^-phendione and AmB (Figure 4, AmB + Cu-p) induced pronounced ultrastructural modifications, such as mitochondrial swelling, lipid accumulation, and the presence of autophagic structures. Overall, the combination of ¼ × IC_50_ AmB with ½ × IC_50_ Cu^2+^-phendione caused more severe morphological changes than either compound alone, suggesting increased cellular damage in *L. amazonensis* promastigotes.

### 3.4. Effects on Plasma Membrane Integrity

**Figure 5:**
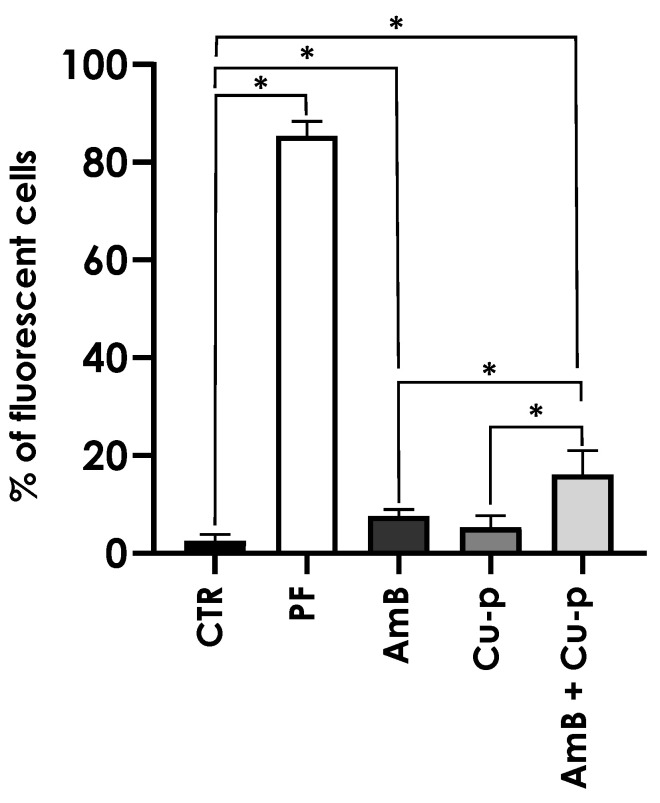
Effects of the combination of ¼ × IC_50_ Amphotericin B (AmB) with ½ × IC_50_ Cu^2+^-phendione (Cu-p) on the plasma membrane permeability of *L. amazonensis* promastigotes. Membrane integrity was assessed by propidium iodide (PI) staining. Parasites were incubated for 48 h in the absence (control—CTR) or presence of ¼ × IC_50_ AmB, ½ × IC_50_ Cu-p, either alone or in combination (AmB + Cu-p). Parasites fixed with 4% paraformaldehyde (PF) were used as a non-viable cell control. Results were expressed as the percentage of fluorescent cells. Data are presented as mean ± standard deviation from three independent experiments performed in triplicate, with 10,000 cells analyzed. Results with *p* < 0.05 (*) were considered significant.

*L. amazonensis* promastigotes were pre-treated for 48 h and subsequently incubated with PI for flow cytometry analysis. Treatment with ¼ × IC_50_ AmB significantly increased the percentage of labeled cells compared with untreated controls. In contrast, ½ × IC_50_ Cu^2+^-phendione alone did not affect membrane permeability. The combined treatment markedly increased PI labeling relative to both single-compound treatments and the control cells, indicating a greater compromise of membrane integrity (Figure 5). Non-viable cells fixed and permeabilized with 4% paraformaldehyde served as a positive control, displaying a high percentage of labeled cells.

### 3.5. Effects on Mitochondrial Metabolism

**Figure 6:**
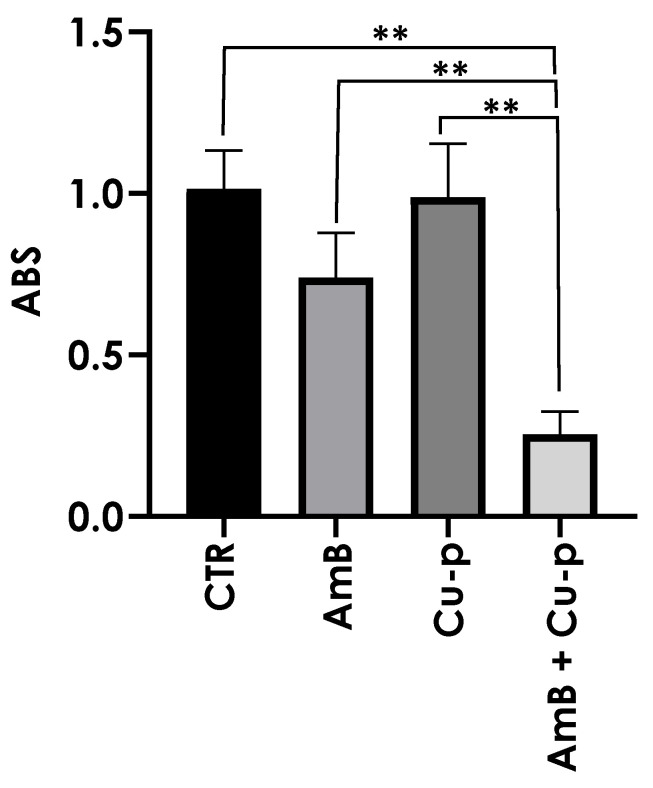
Effects of the combination of ¼ × IC_50_ Amphotericin B (AmB) with ½ × IC_50_ Cu^2+^-phendione (Cu-p) on mitochondrial metabolism in *L. amazonensis* promastigotes. Parasites were incubated for 48 h in the absence (CTR) or presence of ¼ × IC_50_ AmB, ½ × IC_50_ Cu-p, either alone or in combination (AmB + Cu-p). MTT was then added to the cultures, followed by incubation for 3 h at 28 °C. The supernatant was removed, and the resulting formazan crystals were dissolved in DMSO. Absorbance (ABS) was measured at 490 nm using a microplate reader. Data are presented as mean ± standard deviation from three independent experiments performed in triplicate, with 10,000 cells analyzed. Results with *p* < 0.005 (**) were considered significant.

The mitochondrial metabolism of promastigotes under different treatment conditions was assessed using the MTT assay. Treatment with ½ × IC_50_ Cu^2+^-phendione or ¼ × IC_50_ AmB alone did not significantly affect mitochondrial metabolism compared with untreated control cells. In contrast, the combined treatment significantly reduced mitochondrial metabolism, resulting in a 75% reduction relative to all other conditions (Figure 6).

### 3.6. Effects on Mitochondrial Membrane Potential

**Figure 7:**
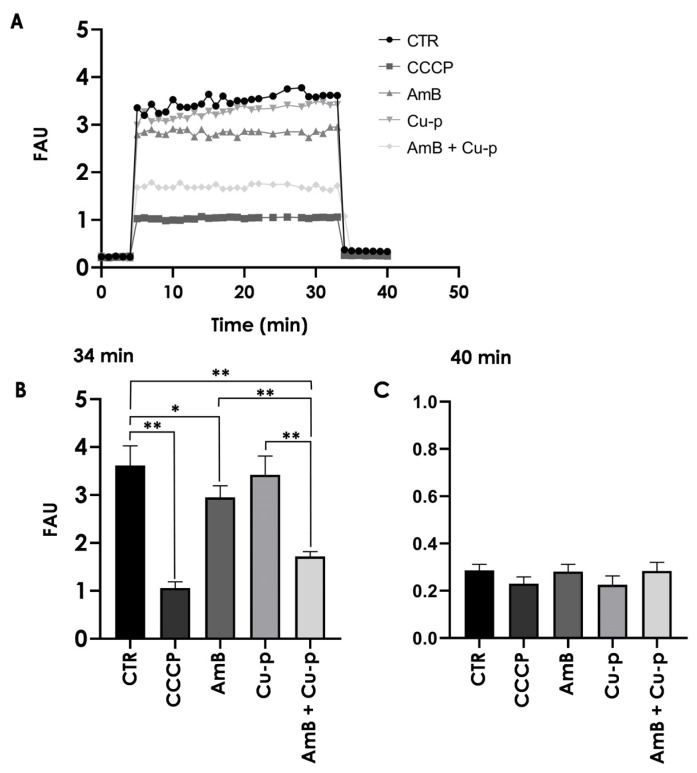
Effects of the combination of ¼ × IC_50_ Amphotericin B (AmB) with ½ × IC_50_ Cu^2+^-phendione (Cu-p) on mitochondrial membrane potential (ΔΨm) in *L. amazonensis* promastigotes. Parasites were incubated for 48 h in the absence (CTR) or presence of ¼ × IC_50_ AmB, ½ × IC_50_ Cu-p, either alone or in combination (AmB + Cu-p). (**A**) ΔΨm was assessed using the JC-1 fluorochrome for 30 min. After this period, the uncoupler CCCP (2 μM) was added to all systems to collapse the mitochondrial potential. As a positive control for depolarization, the reaction was also performed in the presence of CCCP (1 μM) from the beginning of the experiment. (**B**,**C**) Comparison of ΔΨm values shown in (**A**) before (**B**, 34 min) and after (**C**, 40 min) the addition of CCCP. Results are expressed as fluorescence arbitrary units (FAU). Data are presented as mean ± standard deviation from three independent experiments performed in triplicate, with 10,000 cells analyzed. Results with *p* < 0.05 (*) and *p* < 0.001 (**) were considered significant.

Mitochondrial membrane depolarization was evaluated using JC-1 staining. Treatment with ¼ × IC_50_ AmB caused a significant 18% reduction in ΔΨm compared with control cells, an effect not observed with ½ × IC_50_ Cu^2+^-phendione (Figure 7A,B). In contrast, the combined treatment produced a markedly greater decrease in ΔΨm, with a 52% reduction relative to the control and reductions of 41% and 49% compared with AmB and Cu^2+^-phendione alone, respectively (Figure 7A,B). Pre-incubation of parasites with CCCP induced a significant reduction in ΔΨm, and addition of the mitochondrial protonophore after 34 minutes resulted in complete mitochondrial membrane depolarization in all systems (Figure 7C).

### 3.7. Effects on Reactive Oxygen Species (ROS) Production

**Figure 8:**
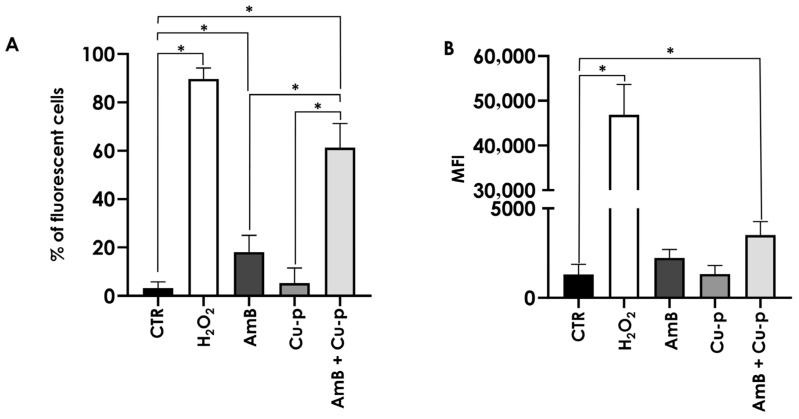
Effects of the combination of ¼ × IC_50_ Amphotericin B (AmB) with ½ × IC_50_ Cu^2+^-phendione (Cu-p) on ROS induction in *L. amazonensis* promastigotes. Parasites were incubated for 48 h in the absence (control—CTR) or presence of ¼ × IC_50_ AmB, ½ × IC_50_ Cu-p, either alone or in combination (AmB + Cu-p). ROS production was assessed by incubation with the H_2_DCFDA probe and analyzed by flow cytometry. Parasites treated with H_2_O_2_ were used as a positive control for ROS production. Results were expressed as the percentage of fluorescent cells (**A**) and mean fluorescence intensity (MFI) (**B**). Data are presented as mean ± standard deviation from three independent experiments performed in triplicate, with 10,000 cells analyzed. Results with *p* < 0.05 (*) were considered significant.

ROS production was evaluated using the H_2_DCFDA probe. Flow cytometry analysis showed that treatment with ½ × IC_50_ Cu^2+^-phendione did not induce ROS generation, whereas promastigotes treated with ¼ × IC_50_ AmB exhibited a 5.6-fold increase in the percentage of fluorescent cells compared with untreated control. Notably, the combination of both compounds resulted in an 18.8-fold increase relative to the control, corresponding to an approximately 3.4-fold increase compared with AmB treatment alone (Figure 8A). Regarding mean fluorescence intensity, only parasites exposed to the combined treatment displayed a significant 2.5-fold increase relative to the untreated controls. Cells incubated with H_2_O_2_ served as a positive control for ROS generation (Figure 8B).

### 3.8. Effects on the Adhesion of Promastigotes to THP-1 Cells

We also evaluated whether the combination of ¼ × IC_50_ AmB and ½ × IC_50_ Cu^2+^-phendione could modulate the adhesion/interaction process with host cells, which represents the first step in infection establishment. For this purpose, *L. amazonensis* promastigotes pre-treated with the compounds, individually or in combination, were incubated with THP-1 cells at a parasite-to-cell ratio of 10:1, at 37 °C for 24 h. It is worth mentioning that this combination did not affect THP-1 macrophages viability (Figure S1).

**Figure 9:**
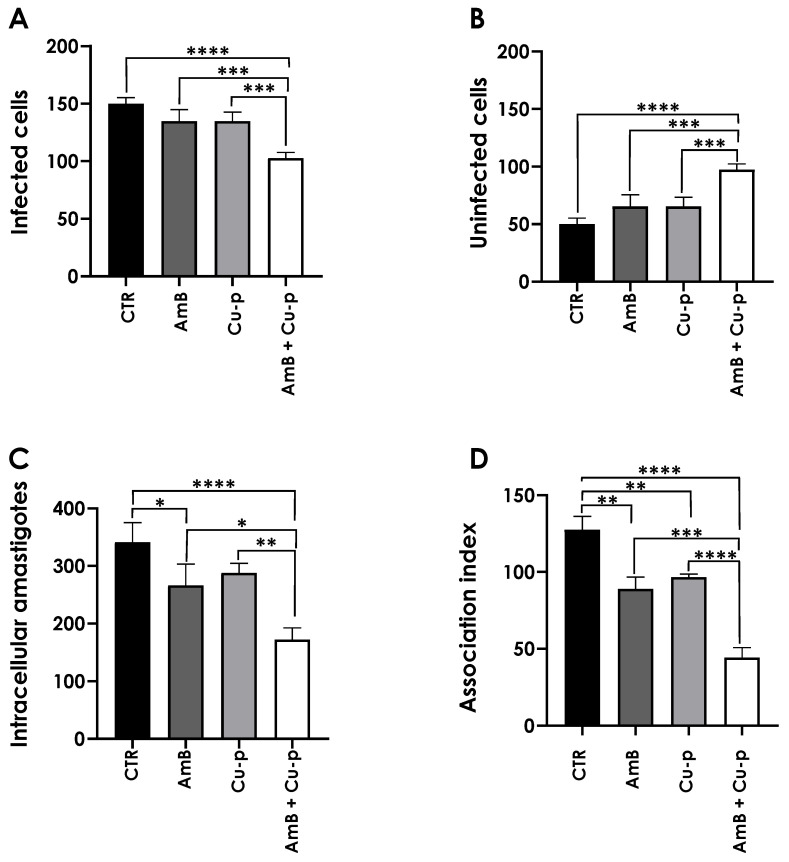
Effects of pre-treatment of *L. amazonensis* promastigotes with the combination of ¼ × IC_50_ Amphotericin B (AmB) with ½ × IC_50_ Cu^2+^-phendione (Cu-p) on the interaction with THP-1 mammalian cells. Parasites were pre-treated or not (CTR) for 4 h with ¼ × IC_50_ Amphotericin B (AmB) and ½ × IC_50_ Cu^2+^-phendione (Cu-p), either alone or in combination. Subsequently, parasites were incubated with THP-1 cells at a parasite/host cell ratio of 10:1 at 37 °C for 24 h. After incubation, cells were Giemsa-stained, and 200 cells were counted per slide in triplicate using light microscopy. Graphs show the number of infected cells (**A**), non-infected cells (**B**), intracellular amastigotes (**C**), and the association index (**D**). Data are presented as mean ± standard deviation from three independent experiments performed in triplicate, with 10,000 cells analyzed. Results with *p* < 0.05 (*), *p* < 0.005 (**), *p* < 0.001 (***) and *p* < 0.0001 (****) were considered significant.

In this set of experiments, the impact of pre-treatment on promastigotes infectivity was assessed by quantifying the intracellular development of amastigotes, which occurs only when parasites are able to adhere to and invade macrophages. Figure 9A shows the number of infected THP-1 cells. Promastigotes pre-treated with the combination of compounds exhibited a 32% reduction in the number of infected cells compared to the untreated control, whereas pre-treatment with each compound alone produced no significant effect. In contrast, the number of non-infected cells remained similar between the control and each individual treatment, while the combined treatment led to a 94% increase in non-infected cells relative to the control (Figure 9B). As shown Figure 9C, the number of intracellular amastigotes was significantly lower in the system treated with the combination compared to both the control and the individual treatments. Treatment with ¼ × IC_50_ AmB reduced the number of intracellular amastigotes by approximately 22% relative to the control, whereas Cu^2+^-phendione alone had no significant effect. In contrast, the combined treatment was more effective, reducing amastigote replication by 49% compared to the control (Figure 9C). Notably, the combination decreased the number of intracellular amastigotes by 1.5- and 1.6-fold compared to the treatment with AmB and Cu^2+^-phendione alone, respectively (Figure 9C). The association index, shown in Figure 9D, was calculated by multiplying the percentage of infected cells by the number of amastigotes per infected cell. Treatment with the individual fractions of AmB and Cu^2+^-phendione reduced the association index by 30% and 24%, respectively, compared to the control. However, pre-treatment with the combination of the drugs resulted in a 65% reduction relative to the control. Moreover, the decrease in the association index following the combined treatment was significantly greater than that observed for each compound alone, corresponding to reductions of 49% compared to AmB and 45% compared to Cu^2+^-phendione.

### 3.9. Type of Interaction of AmB and Cu^2+^-Phendione

**Figure 10:**
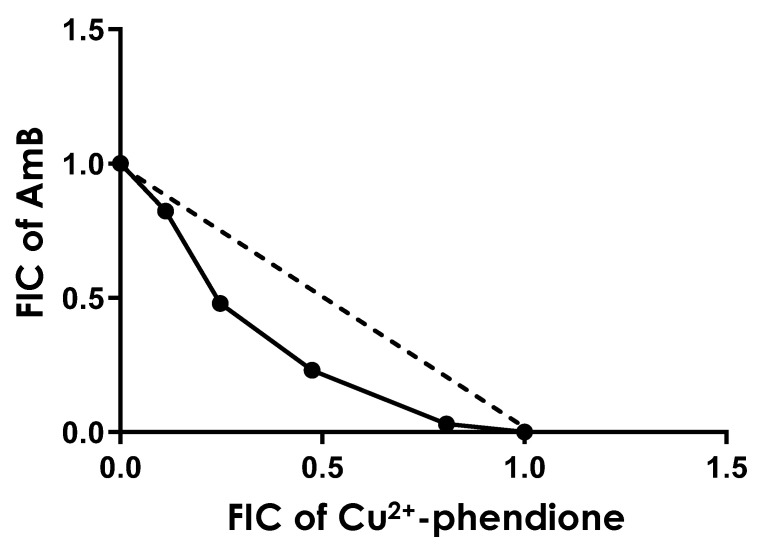
Isobologram analysis of the combination of Cu^2+^-phendione and Amphotericin B (AmB) in different ratios against *L. amazonensis* promastigotes. The plotted points in the isobologram are the fractional inhibitory concentrations (FICs) of each compound in each combination, as presented in Table 1. The straight dashed line represents the theoretical line of additivity for each combination.

*L. amazonensis* promastigotes were incubated with varying ratios of AmB and Cu^2+^-phendione (5:0, 4:1, 3:2, 2:3, 1:4, and 0:5), as described in Section 2. The calculated IC_50_ values, along with the FIC and ΣFIC for each combination, are presented in Table 1. The mean ΣFIC was 0.80, indicating an additive interaction. However, the isobologram analysis (Figure 10) revealed ratio-dependent effects: the 2:3 and 3:2 combinations approached synergism, whereas the 1:4 and 4:1 ratios were consistent with additivity.

## 4. Discussion

Despite its severity, leishmaniasis receives limited investment from the pharmaceutical industry, largely due to its low profitability, as the disease primarily affects low-income populations [40,41]. In this context, the development of more effective and safer therapeutic alternatives is essential, including innovative compounds and novel treatment strategies, such as combination therapies. The present study evaluated the combined effect of Amphotericin B (AmB), a reference drug for leishmaniasis treatment, with Cu^2+^-phendione against *L. amazonensis*, whose antiparasitic activity has been previously reported [14]. This approach leverages two compounds with distinct mechanisms of action, potentially enabling dose reduction and shorter treatment regimens [17,42]. Previous cytotoxicity studies have shown that Cu^2+^-phendione is well tolerated in vitro by several mammalian tumor and non-tumor cell lines, as well as by macrophages, and in vivo by Swiss mice and *Galleria mellonella* larvae [43,44]. Its high selectivity, multi-target activity, low cost, and simple synthesis [43] underscore its potential as a leishmanicidal agent and support its use in combination with AmB to enhance efficacy and reduce adverse effects, thereby improving patient adherence.

In the present study, our results showed that the combination of ¼ × IC_50_ AmB and ½ × IC_50_ Cu^2+^-phendione caused a significant decrease in parasite survival at all time intervals, up to 72 h of treatment, in comparison to control cells. Morphological alterations were also observed, including reduced cell size, body rounding, and flagellum shortening or loss. Transmission electron microscopy allowed the identification of the mechanisms of action associated with different treatments. The isolated Cu^2+^-phendione fraction did not induce ultrastructural changes in the parasite, whereas AmB promoted a significant accumulation of intracellular lipids. A similar pattern of lipid accumulation was observed in cells treated with the combination of these compounds. These observations may indicate an association with mitochondrial dysfunction or oxidative stress, as suggested by previous studies showing that antiparasitic drugs can act by interfering with lipid metabolism or inhibiting mitochondrial activity, thereby inducing lipid droplet formation in trypanosomatids [45]. The accumulation of lipids in lipid droplets is consistent with a defense strategy of the parasites against oxidative stress, storing oxidized lipids and preserving cellular integrity. Lipid droplets may also act as sterol reservoirs, and inhibition of sterol biosynthesis combined with pro-oxidant drugs has been reported to increase parasite susceptibility and enhance antiparasitic efficacy [46]. Although our study did not directly test these mechanisms, the similarities in lipid accumulation patterns suggest that related processes might be occurring here as well. Furthermore, we also observed structures compatible with autophagosomes in parasites treated with the compound combination. In *Leishmania* spp., autophagy is known to be essential for life cycle progression and for responding to stress stimuli, such as nutrient deprivation, oxidative stress, and drug exposure. Inhibition of autophagy under stress conditions increases cell mortality, underscoring its protective role [47]. In our study, the detection of autophagic-like structures within the mitochondrion of *L. amazonensis* may suggest that mitochondrial stress contributes to the activation of this pathway. Previous studies in *Leishmania donovani* demonstrated that autophagy, particularly involving Atg8 protein, plays a central role in survival, infectivity, and differentiation, and that mitochondrial stress and ROS production can lead to the accumulation of Atg8-positive autophagosomes near compromised mitochondria [47]. Although our data do not allow us to assert mechanistic equivalence, the mitochondrial alterations observed here are consistent with the possibility that similar stress-response pathways may be involved. Taken together, our findings, in conjunction with the existing literature, suggest that mitochondrial homeostasis, oxidative stress, and autophagy-related processes could contribute to the parasite’s response to these treatments; however, further functional assays would be required to confirm these mechanisms.

Plasma membrane integrity appeared to be more affected by the combination of Cu^2+^-phendione and AmB than by the individual compounds, as assessed by PI staining. While only a fraction of promastigotes was labeled with ¼ × IC_50_ AmB, the combination was associated with a higher proportion of compromised cells, a pattern resembling the increase previously reported for the combination of AmB with the calpain inhibitor MDL28170, which increased PI labeling eightfold [31]. Moreover, the combination evaluated in the present work was associated with more pronounced mitochondrial alterations, including a decrease in membrane potential and increased ROS production. Excess ROS is known to potentially induce lipid peroxidation, DNA mutations, protein oxidation, and apoptosis [48,49]. In our assays, ¼ × IC_50_ AmB was sufficient to double ROS production, whereas the combination with Cu^2+^-phendione further increased this effect. The combination was also the only treatment that led to a detectable reduction in mitochondrial metabolism under our experimental conditions, which may suggest that mitochondrion represents a relevant target of the combined treatment. Previous studies have shown that Cu^2+^-phendione can induce mitochondrial membrane depolarization and reduce dehydrogenase activity in a concentration-dependent manner, with effects observed from IC_50_ onward [14]. This is notable because *Leishmania* possesses a single mitochondrion essential for survival, and promastigote forms rely on mitochondrial respiration and oxidative phosphorylation for ATP production [48,50]. Taken together, these observations suggest that the Cu^2+^-phendione/AmB combination may simultaneously affect plasma membrane integrity and mitochondrial function, contributing to the decreased viability observed.

Previous studies have demonstrated that Cu^2+^-phendione can negatively modulate the expression of the metallopeptidase gp63, a surface molecule essential for adhesion, invasion, and establishment of infection in host cells [16,51]. *Leishmania braziliensis* [13] and *L. amazonensis* [16] promastigotes treated with Cu^2+^-phendione exhibited reduced levels of both surface gp63 and its intracellular isoforms, accompanied by a significant decrease in proteolytic activity. This modulation directly impacts parasite–host interactions by impairing the parasite’s ability to associate with host cells. In the present study, we observed that pre-treatment of promastigotes with the compound combination resulted in a marked decrease in the number of infected macrophages, leading to a reduced number of intracellular amastigotes, and, consequently, a lower association index. Notably, treatment with the ¼ × IC_50_ AmB fraction also reduced the number of intracellular amastigotes, and both drugs alone decreased the association index, although to a lesser extent than the combination. Previous work has highlighted the relevance of gp63 during the early stages of parasite–host interaction, showing that promastigotes pre-treated with Cu^2+^-phendione exhibit decreased gp63 expression and proteolytic activity, resulting in reduced interaction with host cells. Additionally, experiments in which *L. amazonensis* promastigotes interacted with THP-1 cells in the presence of soluble gp63 showed a considerable increase in the association index compared with controls lacking the enzyme [16]. Taken together, these findings suggest that because Cu^2+^-phendione can interfere with both the expression and activity of gp63, the effects observed in this study may be directly or indirectly related to the modulation of this metallopeptidase. Since our study focused exclusively on promastigote infectivity, it is important to emphasize that further research is needed to assess the direct effects of AmB/Cu^2+^-phendione combination on established intracellular amastigotes, the developmental forms responsible for sustaining and disseminating the infection in vertebrate hosts.

Finally, our results showed that the combination of Cu^2+^-phendione with AmB exhibited an overall additive effect (ΣFIC = 0.80). However, the isobologram revealed that certain ratios (2:3 and 3:2) approached synergy, as indicated by the concave curve falling below the line of indifference. At these ratios, substantial reductions in IC_50_ values were observed: in the 3:2 (Cu^2+^-phendione:AmB) ratio, IC_50_ values decreased by approximately 50% for Cu^2+^-phendione (from 8.37 nM) and 75% for AmB (from 140 nM); in the 2:3 ratio, reductions were approximately 75% for Cu^2+^-phendione and 50% for AmB. These findings suggest that optimizing the relative concentrations of the compounds could enhance the overall efficacy of the combination.

Drug combinations, particularly those pairing classical agents such as AmB with novel molecules possessing distinct mechanisms of action, represent a promising strategy to increase therapeutic efficacy and reduce the likelihood of resistance. Previous studies have shown that AmB can exhibit variable responses depending on the partner compound. In some cases, marked synergism has been reported; for example, its combination with polyaltinic acid (from *Copaifera* spp.) yielded ΣFIC values of 0.36 and 0.39 for *L. amazonensis* promastigotes and amastigotes, respectively, without inducing cytotoxicity or hemolysis [52]. Similar synergistic effects have been reported with crotamine, a snake venom toxin, which not only enhanced antiparasitic activity but also reduced toxicity in mammalian cells [53]. The same pattern was observed for the benzimidazole derivative TCBZ, whose combination with AmB lowered the IC_50_ by up to 120-fold, with a ΣFIC of 0.25 and a marked increase in the selectivity index [54]. Conversely, not all combinations result in synergy. The combination of tamoxifen and AmB showed an additive effect (ΣFIC of 1.23 and 0.74 for promastigotes and amastigotes, respectively). Nonetheless, in a *L. amazonensis*-infected Balb/c mouse model, the combined treatment significantly reduced lesion size and parasite burden compared with monotherapy [55]. These findings illustrate that the interaction profile of AmB strongly depends on the partner drug and the ratio used, with outcomes ranging from synergistic to purely additive. This emphasizes the importance of systematically exploring different combinations and proportions to identify the most promising strategies for leishmaniasis treatment. Thus, although the Cu^2+^-phendione/AmB combination in this study predominantly exhibited an additive effect, the ratios approaching synergy reinforce the potential of this strategy and the need for further investigations in more complex experimental models.

In conclusion, our study demonstrated that the combination of the coordination compound Cu^2+^-phendione, whose anti-*Leishmania* activity has been previously demonstrated by our research group, with AmB, a drug already used in leishmaniasis therapy, exerts potent and significant effects on the proliferation, ultrastructure, and metabolism of *L. amazonensis* promastigotes, as well as on their association index with THP-1 cells. Although our results indicate an overall additive interaction, the selected combination, ¼ × IC_50_ AmB plus ½ × IC_50_ Cu^2+^-phendione, represents a promising chemotherapeutic alternative. Using lower doses of AmB in combination with Cu^2+^-phendione offers distinct therapeutic advantages. By reducing the required concentration of AmB, the combination helps minimize its major dose-dependent toxicity, thereby improving treatment tolerability and patient safety. Lower AmB doses can also decrease the need for intensive clinical monitoring and reduce hospitalization costs, particularly in settings where liposomal formulations are used. Importantly, Cu^2+^-phendione acts through mechanisms that are partially distinct from those of AmB, a finding that provides a valuable insight into their complementary modes of action and helps explain the enhanced activity observed in the combination despite the reduced AmB concentration. At the same time, the possibility of maintaining or even improving efficacy while reducing AmB exposure highlights the potential of this combination to optimize treatment outcomes while overcoming toxicity-related barriers to patient adherence. Given the antileishmanial activity against *L. amazonensis* promastigotes, further in vivo investigations are necessary to assess the effects of this combination on intramacrophage replicative amastigotes and to better characterize drug interactions, which may provide a basis for future treatment recommendations for different forms of leishmaniasis.

## Figures and Tables

**Table 1:** Determination of FIC and ΣFIC values for the combination of Amphotericin B (AmB) and Cu^2+^-phendione (Cu-p) on promastigotes of *L. amazonensis*.

		IC_50_ (nM)		FIC		∑FIC	∑FIC (Mean)
Cu-p	AmB	Cu-p	AmB	Cu-p	AmB		
5	0	6.53	---				0.8
4	1	5.27	3.2	0.80	0.031	0.83	
3	2	3.10	23.7	0.47	0.23	0.70	
2	3	1.61	49.2	0.24	0.48	0.72	
1	4	0.73	84.6	0.11	0.82	0.93	
0	5	---	102.8				

## Data Availability

The original contributions presented in this study are included in the article/Supplementary Material. Further inquiries can be directed to the corresponding authors.

## Supplementary Materials

The following supporting information can be downloaded at: https://www.mdpi.com/article/10.3390/tropicalmed11010004/s1, Figure S1: Effects of the combination of ¼ × IC_50_ Amphotericin B (AmB) with ½ × IC_50_ Cu^2+^-phendione on the mitochondrial metabolism of THP-1 cells.
