# Supplementary material for: In Vitro Assessment of the Combined Activity of Amphotericin B and Cu2+-1,10-Phenanthroline-5,6-dione Coordination Compound Against Leishmania amazonensis Promastigotes
Source: Trop Med Infect Dis. 2025 Dec 24;11(1):4. doi: 10.3390/tropicalmed11010004 (PMC12846362; doi:10.3390/tropicalmed11010004)
Supplement: Supplementary file 1 [file tropicalmed-11-00004-s001.zip › tropicalmed-4014931-supplementary.pdf]

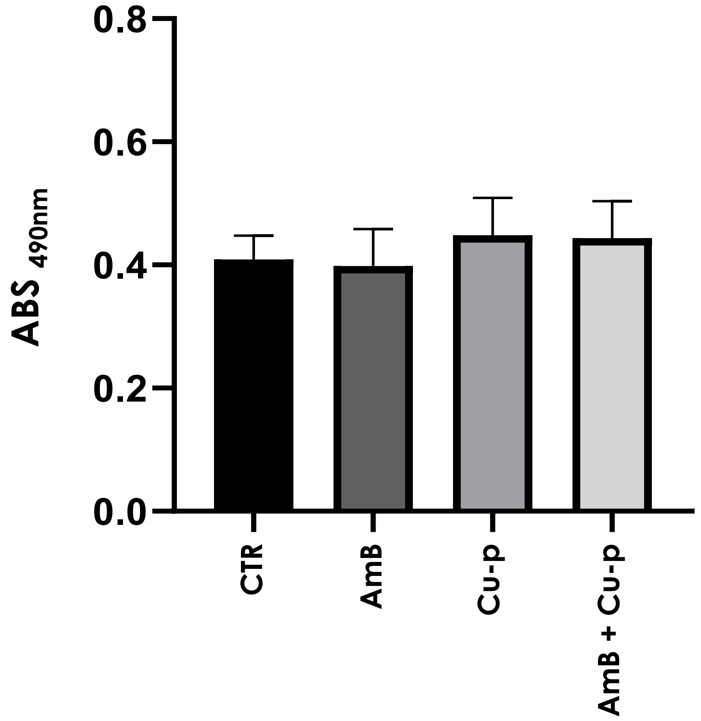

Figure S1. Effects of the combination of ¼ × IC_{50} Amphotericin B (AmB) with ½ × IC_{50} Cu^{2+}-phendione on the mitochondrial metabolism of THP-1 cells. Cells were incubated for 48 h in the absence (CTR) or presence of ¼ × IC_{50} AmB and ½ × IC_{50} Cu-p, either alone or in combination (AmB + Cu-p). MTT was then added to the cultures, followed by incubation for 4 h at 37 °C. The supernatant was removed, and the resulting formazan crystals were dissolved in DMSO. Absorbance (ABS) was measured at 490 nm using a microplate reader. Data are presented as mean ± standard deviation from three independent experiments performed in triplicate.
